# High probability of successive occurrence of Nankai megathrust earthquakes

**DOI:** 10.1038/s41598-022-26455-w

**Published:** 2023-01-10

**Authors:** Yo Fukushima, Tomoaki Nishikawa, Yasuyuki Kano

**Affiliations:** 1 International Research Institute of Disaster Science, Tohoku University, Sendai, Japan; 2 Disaster Prevention Research Institute, Kyoto University, Uji, Japan; 3 Earthquake Research Institute, University of Tokyo, Tokyo, Japan

**Keywords:** Natural hazards, Seismology

## Abstract

Great earthquakes along the Nankai megathrust in south-western Japan feature in the top priority list of Japan’s disaster management agenda. In May 2019, an alert system was incepted to issue public warnings when the probability of an earthquake occurrence along the Nankai megathrust became higher than usual. One of the cases that trigger the issuance of public warnings is when a great earthquake occurred and another one of the same scale is anticipated within a short period of time. Although such “twin ruptures” have occurred multiple times along the Nankai megathrust, the quantification of the probability of such twin ruptures has never been attempted. Based on global statistics and local earthquake occurrence history, we estimated the probability of a successive occurrence of two M8 or larger earthquakes within 3 years globally and along the Nankai megathrust to be 5.3–18% and 4.3–96%, respectively. The timing of the second earthquake followed the Omori–Utsu law in global statistics, which allowed the estimation of the probability for the successive occurrence of Nankai megathrust earthquakes in arbitrary time frames. The predicted probability for the one-week timeframe was 100–3600-fold higher than that of the norm, endorsing the necessity for the warning scheme.

## Introduction

The methods of probabilistic earthquake forecasts have been implemented in a number of earthquake-prone countries^1^. In Japan, the Earthquake Research Committee (ERC) of the Headquarters of Earthquake Research Promotion of Japan provides long-term (commonly 30 to 100 years) evaluations of the occurrence potentials of both inland and subduction-zone earthquakes in Japan^2^, which are then used for the creation of the probabilistic seismic hazard map^3^. The Uniform California Earthquake Rupture Forecast, Version 3 (UCERF3), is the latest official earthquake rupture forecast of the state of California in the United States, providing authoritative estimates of the probability of long-term fault rupture as well as the short-term probability of aftershocks^4^. In New Zealand, GeoNet, the official source of geological hazard information in New Zealand, provides time-varying probabilities of earthquake occurrence^5^. These operational earthquake forecasts mainly focus on either long-term probabilities or short-term aftershock probabilities. Operational short-term forecasting of great earthquakes has still been challenging.

**Figure 1:**
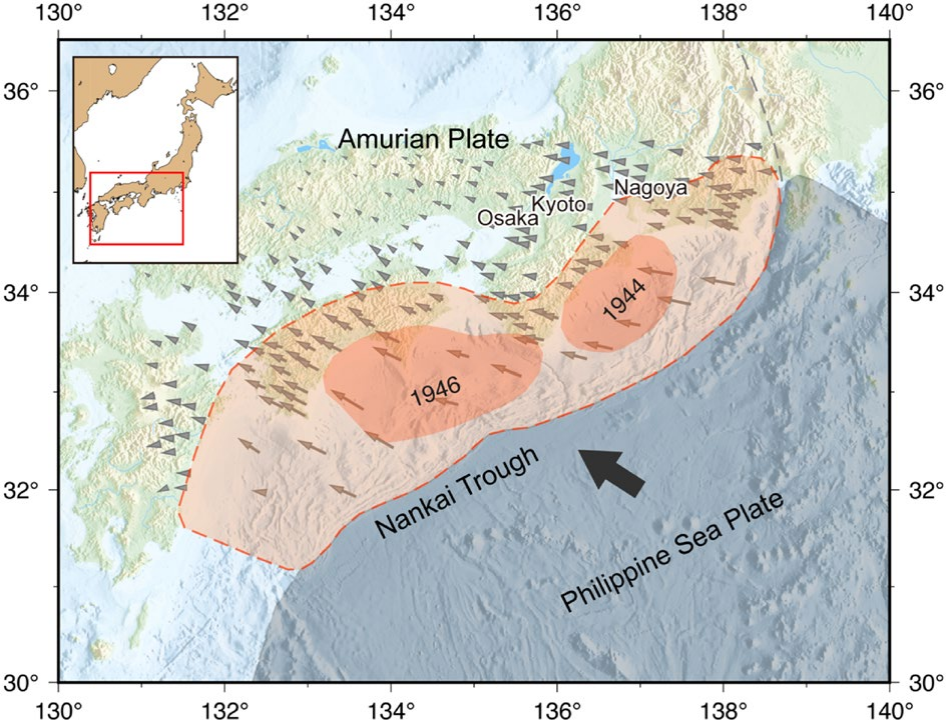
Tectonic setting of the Nankai Trough megathrust. The grey area indicates the Philippine Sea Plate. The thick arrow indicates the plate convergence direction. The grey arrows indicate ground displacements^12^. The light orange area corresponds to the maximum-possible fault area published by the Japanese Government.

In south-western Japan, great earthquakes of magnitudes 8.0–8.7 have occurred repeatedly at intervals of 100 to 150 years, causing severe damage^6–11^. These earthquakes commonly rupture the megathrust fault that separates the overriding Amurian Plate from the Philippine Sea Plate, subducting from the Nankai Trough (Fig. 1). As predicted using the megathrust locking distribution, similar earthquakes will occur along the Nankai megathrust in the future^12^. The ERC estimates that the 30-year occurrence probability of a great Nankai megathrust earthquake is 70–80% as of January 2022^2^. Although this could be an overestimate^13^, a range of calculations based on different statistical models and assumptions of the average recurrence interval whilst considering the earthquake history after the fourteenth century has given a 30-year occurrence probability of 5–80%^2^. Despite the significant uncertainty, the need for reducing the disaster risk is clear considering the adjacent populated and industrialized areas, including the metropolitan cities of Osaka, Kyoto, and Nagoya. Accordingly, the great Nankai megathrust earthquakes feature in the top priority list of Japan’s disaster management strategy.

The great Nankai megathrust earthquakes have often occurred as “twin” ruptures, *i.e.*, two M8-class earthquakes occurring successively within an interval of 2 years^6,14,15^. The 1854 twin earthquakes successively ruptured the fault areas east and west of the Kii Peninsula around 136°E within a 30-h interval. The 1944 and 1946 twin earthquakes ruptured smaller and slightly different fault areas than those ruptured by the 1854 twin earthquakes (Fig. 1)^6,16,17^. Such twin ruptures are presumed intrinsic to the nature of the Nankai megathrust.

For over a decade, slow slip events (SSEs) have preceded great subduction zone earthquakes on multiple occasions^18–24^. For example, the Tohoku-Oki earthquake of moment magnitude (Mw) 9.0 in north-eastern Japan that occurred in March 2011 was preceded by an SSE, which had started a month before^18^, associated with foreshock migrations^19^. As SSEs are abundant along the Nankai megathrust, their use in earthquake forecasting has been discussed actively^25,26^. These led to a government study group to state “We can qualitatively regard, though with uncertainty, that the probability of earthquake occurrence has become higher than normal when the state of plate locking is changed, for example by expansion or acceleration of a slow slip” (translated to English by the authors)^27^.

The high probability of an earthquake occurrence, its social impacts, historical evidence of the successive occurrence of past Nankai megathrust earthquakes, and the discovery of precursory SSEs worldwide have prompted the Japanese Government to introduce a new warning scheme in May 2019 against forthcoming great Nankai megathrust earthquakes. Under this scheme, the Japan Meteorological Agency warns the public when the probability of a great Nankai megathrust earthquake increases to a level exceeding the norm. An earthquake of Mw 7.0 or larger around the megathrust and an abnormal slow slip on the megathrust can trigger these warnings.

A major drawback of this scheme is that a warning is not accompanied by the probability of earthquake occurrence. This constrains appropriate public response to the threat as, conventionally, decision making is based on the chance of an event occurring and its consequences. Given the current knowledge in seismology, accurately predicting a future event is not possible. However, predicting likely scenarios and their rough probability rates could assist the society in formulating countermeasures and responding effectively to the warnings. Considering the history of multiple twin ruptures, the successive occurrence of M8-class earthquakes is a likely and most concerning case for the Nankai megathrust. Therefore, probability estimation for such an occurrence is a particular need of the society. Here, we aimed to estimate the probability of successive great earthquake occurrence along the Nankai megathrust.

## Results

### Probability of successive earthquakes based on global catalogues

On the basis of global earthquake statistics, we first inferred the probabilities of successive occurrence of an earthquake of Mw 7.0 or larger and an earthquake of Mw 8.0 or larger. This was conducted by independent verification and elaboration of the findings of a previous study^28^, which were reflected in the official guidelines for disaster management measures in response to warnings^29^.

We referred to earthquakes of Mw larger than or equal to 8.0 as “M8 + earthquakes”. Similarly, we referred to earthquakes of Mw larger than or equal to 7.0 and smaller than 8.0 as “M7-class earthquakes”. We calculated the probabilities of an M7-class or M8 + earthquake, followed by another M8 + earthquake nearby, along with their probability gains and confidence intervals (see “Methods” for details).

We used two global earthquake catalogues, namely the International Seismological Centre-Global Earthquake Model (ISC-GEM ver 6.0) Global Instrumental Earthquake Catalogue^30,31^ and the Advanced National Seismic System (ANSS) Comprehensive Earthquake Catalog^32^. Inclusion criteria for these data sets were as follows: (1) earthquakes in all regions without area restrictions and (2) subduction zone earthquakes only. A previously reported approach was adopted^28^ with modifications. The previous study used only the ISC-GEM ver 6.0 Global Instrumental Earthquake Catalogue, did not assess restricting the area to subduction zones, and did not evaluate the uncertainties.

The probabilities calculated using the ISC-GEM ver 6.0 Global Instrumental Earthquake Catalogue and ANSS Comprehensive Earthquake Catalog data sets for all the regions and subduction zones were consistent (Supplementary Tables S1 and S3). Subsequently, the results obtained using the ISC-GEM ver 6.0 Global Instrumental Earthquake Catalogue without area restrictions were used.

Among the 105 M8 + events recorded from 1904 to 2015, the number of subsequent M8 + earthquakes that occurred in the vicinity within 1 day, 3 days, 1 week, 2 weeks, and 3 years were 2, 3, 3, 5 and 11 (1.9, 2.9, 2.9, 4.8 and 10%), respectively. The confidence intervals were usually wide (0.23–6.7% within 1 day, 1.6–11% within 1 week, and 5.3–18% within 3 years) owing to the small sample size (Supplementary Table S1).

Among the 1,354 M7-class events recorded from 1904 to 2015, the number of subsequent M8 + earthquakes that occurred in the vicinity within 1 day, 3 days, 1 week, 2 weeks, and 3 years were 3, 5, 8, 9 and 23 (0.22, 0.37, 0.59, 0.66 and 1.7%), respectively (Supplementary Table S3). The confidence intervals were narrower than those of successive M8 + cases, ascribed to the relatively large sample size. Moreover, the probability of an “M7 class–M8 + successive occurrence” was smaller by one order than that of an M8 + successive occurrence.

Considering the confidence intervals, the results were consistent with the official guidelines of countermeasures in response to a warning of Nankai megathrust earthquakes^29^. The guidelines state that, “the frequency of an M8-class or larger earthquake occurring within 7 days after an earthquake of M8.0 or larger is once per just over a dozen times”, and “the frequency of an M8-class or larger earthquake occurring within 7 days after an earthquake of M7.0 or larger is once per a few hundred times.”

The probability gains, obtained by dividing the probability of successive occurrence by the base rate, are listed in Supplementary Tables S4 and S5. Here, we calculated the base rates of probability by assuming a Poisson model, with an average recurrence interval of 90 years (Supplementary Table S2), which applies specifically to the Nankai Trough region (see “Methods” for details of the calculation).

As expected, we observed a sharp rise in the probability gains just after the occurrence of the first earthquake. For example, the probability of a successive M8 + occurrence within a day was 76 to 2200 times higher than usual, and the probability gain dropped to 1.6 to 5.5 in 3 years (Supplementary Table S4). Slight differences were observed between the probability gains in the current study and those stipulated in the guidelines^29^. However, we do not discuss the differences in detail as they do not have crucial implications for our conclusions.

**Figure 2:**
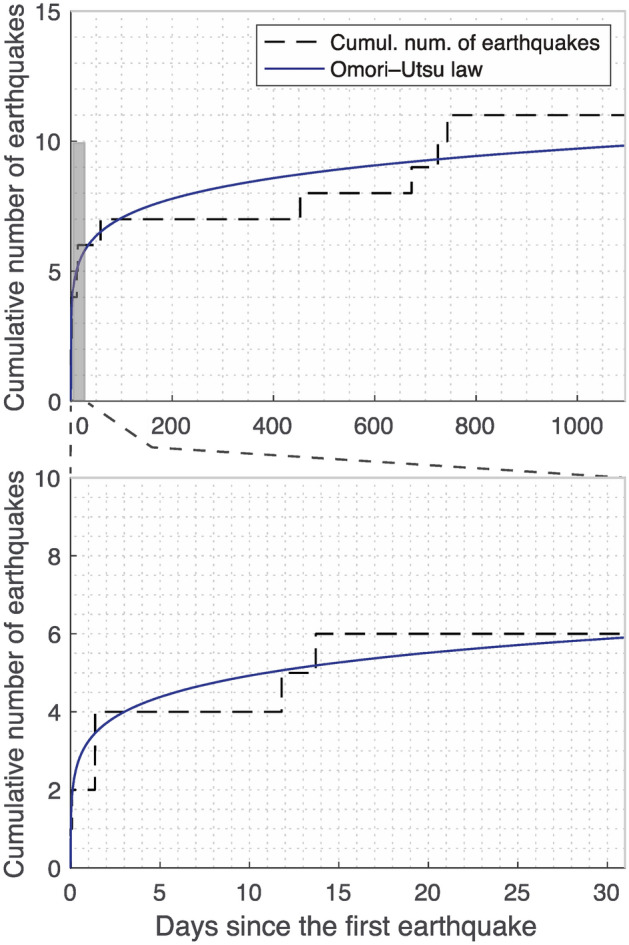
Cumulative number of earthquakes with respect to time. The data set of the Seismological Centre-Global Earthquake ver 6.0 Global Instrumental Earthquake Catalogue was used without regional restrictions. The cumulative number of earthquakes is demonstrated by the stair plot. The best-fit curve of the Omori–Utsu law is shown in blue.

The cumulative count of earthquakes with respect to time aligned with the predictions obtained using the Omori–Utsu law used for modelling the occurrence rate of aftershocks of a single earthquake (Fig. 2). In the Omori–Utsu law, the aftershock rate $n\left(t\right)$ and the cumulative number of aftershocks $N(t)$ are expressed by

1 $$\begin{array}{c}n\left(t\right)= {K\left(t+c\right)}^{-p} ,\end{array}$$

an﻿d

2 $$\begin{array}{c}N\left(t\right)= {K\{{c}^{1-p}-\left( t+c\right)}^{1-p}\} / (p-1), \; \left(p\ne 1\right),\end{array}$$

where $K, c, p$ are constants. The best-fit values of the constants are $K=0.65$, $c=1.0\times {10}^{-3}$, and $p=0.90$. As our findings are consistent with the Omori–Utsu law, we considered the aftershock law suitable for modelling the probability of the successive occurrence of similar-size earthquakes.

## Probability of successive occurrence from past Nankai megathrust earthquakes

Subsequently, we focused on the “twin earthquake” scenario along the Nankai megathrust, where an M8-class earthquake is followed by an event of the same scale, which has the potential to have the most momentous impact on the society.

The history of Nankai megathrust earthquakes has been extensively studied using historical documents and archaeological and geological surveys^7–9,11,14,33–35^. Here, we first elucidated the rupture segments of past earthquakes by reviewing previous studies on past earthquakes (see “Methods”). We focused on the earthquakes that occurred in 1361 and later, which were understood relatively well compared with earlier earthquakes. All the earthquakes discussed below are inferred to be larger than Mw 8.0 by previous studies^6,16,17,35–41^ with an exception (M7.9) from one study for the 1605 earthquake (Supplementary Table S6), and were categorized as M8 + earthquakes in this study.

**Figure 3:**
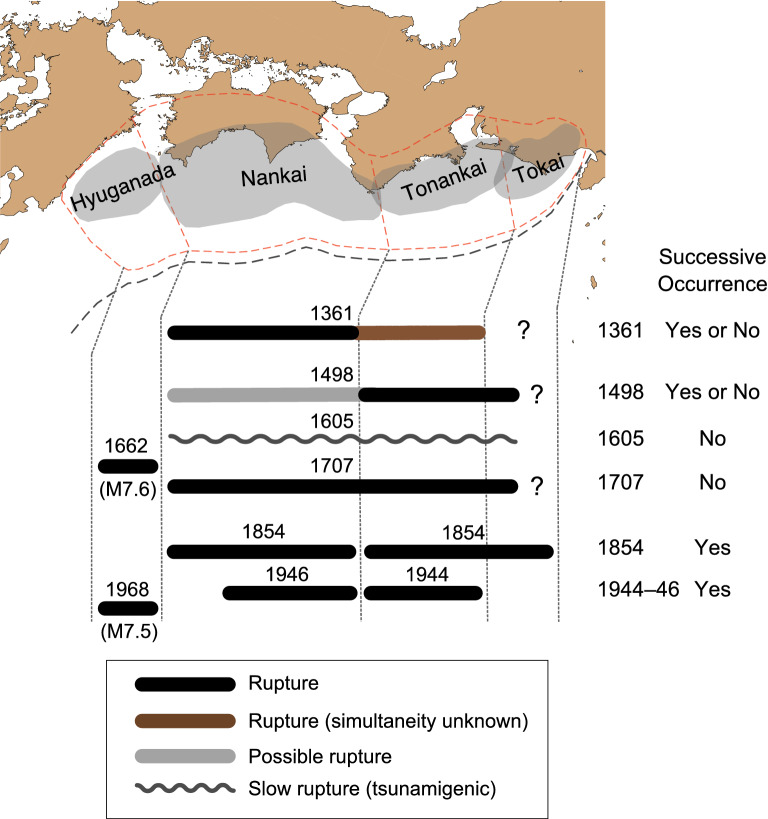
Extent of rupture of past Nankai megathrust earthquakes. The black and brown bars indicate the approximate extent of rupture in the indicated years. The grey bar indicates either separate rupture, simultaneous rupture with the eastern segments, or no rupture. The question marks indicate that the rupture extent is unconstrained.

The results of our re-evaluation of the historical events are summarized in Fig. 3. In association with the warnings for Nankai megathrust earthquakes, the focus here is on the percentage of twin earthquake cases, where two M8 + earthquakes occur successively within a short time interval. Among the earthquake sequence of 1361, 1498, 1605, 1707, 1854 and 1944–1946, the evident successive M8 + earthquakes (twin earthquakes) occurred in 1854 and 1944–1946. Possible successive M8 + earthquake cases occurred in 1361 and 1498. A single M8 + earthquake was observed in 1605 and 1707. The probability (more precisely, the maximum likelihood estimates of the probability) of successive M8 + earthquake occurrences within 3 years was 33% (two out of six) or 67% (four out of six), depending on how the relevant cases were counted. The 95% confidence intervals for the two cases were 4.3–77% and 22–96%, respectively. Combining the two cases, the confidence interval was 4.3–96%. The 3-year probability of successive M8 + earthquakes for the Nankai megathrust was higher than that of the global average (10%, with 95% confidence interval of 5.3–18%), although the confidence intervals overlapped.

## Probability curve for occurrence of successive Nankai megathrust earthquakes

As shown earlier, the occurrence of an M8 + earthquake followed by a subsequent M8 + event is well characterized by the Omori–Utsu law. Here, we assumed that the successive occurrence of Nankai megathrust earthquakes follow the Omori–Utsu law, with $c=1.0\times {10}^{-3}$ and $p=0.90,$ derived from global statistics. The cumulative probability for a successive occurrence of Nankai megathrust earthquakes can be written as follows:

3 $$\begin{array}{c}P\left(t\right)=1-\mathrm{exp}\left(-N\left(t\right)\right),\end{array}$$

assuming a non-stationary Poisson process and considering that the cumulative number $N(t)$ (Eq. (2)) is the transformed time^42^. The non-stationary Poisson process has been assumed in the aftershock occurrence modeling and shown to adequately explain the temporal change of aftershock occurrence rates^42^. The constant $K$ was scaled such that the 3-year probability was 4.3–96% in a 95% confidence interval, consistent with the history of M8 + earthquake occurrence along the Nankai megathrust.

**Figure 4:**
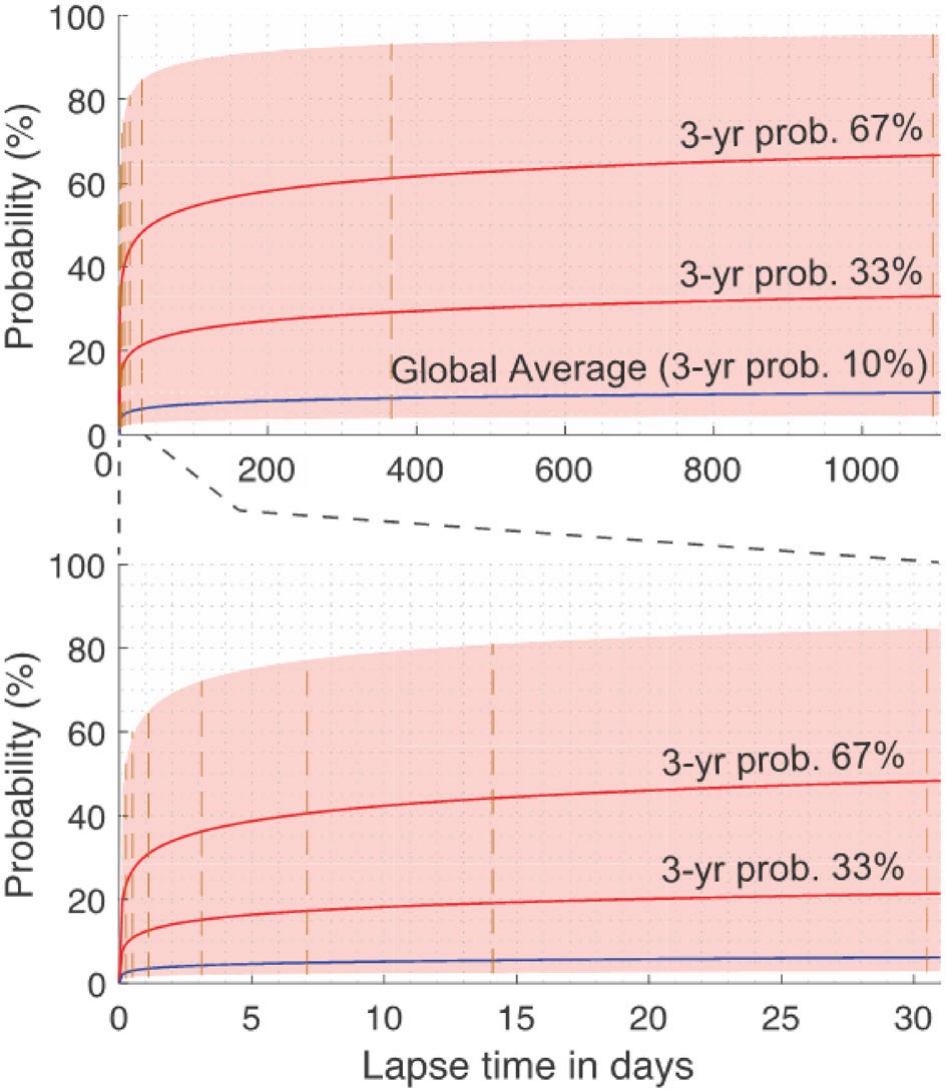
Cumulative probability curve for successive occurrence of great Nankai megathrust earthquakes. Two red curves show the cumulative probability corresponding to 3-year probabilities of 33 and 67%. The red-shaded area shows the confidence interval; minimum and maximum limits corresponding to 3-year probabilities of 4.3 and 96%. The blue curve corresponds to the cumulative probability from global statistics, with 3-year probability of 10%. The vertical broken lines in orange indicate the 95% confidence intervals for different time frames (Table 1).

The resulting probability curves and 95% confidence intervals for the Nankai megathrust are shown in Fig. 4. The probability curve based on the global statistics was near the minimum limit of the confidence interval, implying that the Nankai subduction likely hosts successive M8 + earthquakes more often than the global average indicates. Deriving the confidence interval of the probability curve aided the deduction of the probabilities in arbitrary time frames (Table 1). For example, the probability of an M8 + earthquake occurring within 1 day (24 h) and 1 week after an M8 + earthquake along the Nankai megathrust was 1.4–64% and 2.1–77%, respectively.

**Table 1:** Probability and probability gain for successive occurrence of great Nankai megathrust earthquakes within different time frames.

Time frame	Probability (%)*	Probability gain
6 h	1.0–53	1.3 × 10^3^–7.0 × 10^4^
12 h	1.3–60	8.6 × 10^2^–4.0 × 10^4^
24 h	1.4–64	4.6 × 10^2^–2.1 × 10^4^
3 days	1.8–72	2.0 × 10^2^–7.9 × 10^3^
1 week	2.1–77	99–3.6 × 10^3^
2 weeks	2.3–81	54–2.0 × 10^3^
1 month	2.6–85	28–9.1 × 10^2^
3 years	4.3–96	1.3–29

*Probability of a subsequence earthquake to occur within a designated time frame after the occurrence of the first earthquake.

The probability gains corresponding to the estimated probabilities indicated a sharp rise in probability compared with the norm (Table 1). The probability gains for 6 h was 1000–70,000-fold, decreasing to 100–3600-fold for 1 week and, eventually, to values comparable with the norm (1.3–29) for 3 years.

## Discussion

We verified the appropriateness of the indicative probability for the occurrence of a successive M8 + earthquakes stated in the Japanese Government guidelines^29^, using both similar and different datasets. The 95% confidence intervals in this study clarified the large uncertainties even in the 100-year global datasets. Moreover, the probability of successive occurrence of an M7-class and an M8 + earthquake was one order less than that of successive M8 + earthquakes (Supplementary Tables S1 and S2). This is consistent with expectations from the Epidemic-Type Aftershock Sequence (ETAS) model^43^, which is a standard statistical model of seismicity. Note that the actual probability along the Nankai megathrust for the former remains uncertain because M7-class earthquakes seldom occur along the Nankai megathrust and a similar approach for M8 + earthquakes could not be considered.

An essential aspect of the analysis of global statistics was that the temporal dependency of the frequency of M8 + earthquakes that occur after an M8 + event could be obtained using the Omori–Utsu law (Fig. 3). This may not be surprising because the law describes the temporal decay of the number of aftershocks and an M8 + earthquake following a former one can be regarded as an aftershock. However, this finding was crucial in determining the probability curves of the successive occurrence of M8 + earthquakes.

The 3-year probability estimated from the Nankai megathrust history (4.3%–96%) may indicate high probability of successive Nankai megathrust earthquakes in short time intervals compared to the global average (5.3%–18%). In particular, if we took the “four-out-of-six” case (Section “Probability of successive occurrence from past Nankai megathrust earthquakes”), the 3-year probability (22–96%) is significantly higher than the global average. The wide confidence intervals shown in Table 1 are ascribed to the limited number of known earthquakes along the Nankai megathrust. Nevertheless, the probability table provides essential information for stakeholders and the public.

The rise in the probability from 100–3600-fold for 1 week (probability gain of 99–3.6 × 10^3^, Table 1) justifies the basic policy of the guidelines, including the evacuation of citizens from high-risk areas as a precaution for tsunami events^29^. Equally importantly, the sharp rise in the cumulative probability for 24 h to a few days (Fig. 4, bottom) indicates that, after an M8 + earthquake, immediate and maximum precautions should be implemented against the next event, while responding urgently to the first one. The earthquake emergency plans put in place by the communities likely to be affected by such events must be developed considering the possibility of an immediate occurrence of a successive earthquake. Therefore, responders and the communities should be trained to deal with multiple tremblors.

The wealth of historical documents and multi-disciplinary studies on the Nankai megathrust earthquakes enabled us to derive the probability of successive occurrence of great earthquakes along the megathrust, although the uncertainty was large. In other parts of the world, it is not certain whether successive ruptures result from the intrinsic nature of the area or are simply coincidental. Nevertheless, it is prudent to alert the public to earthquakes that often exhibit a cascade of multiple events.

## Methods

### Calculation of probability of successive occurrence of earthquakes based on global catalogues

We used two global earthquake data sets, namely the ISC-GEM ver 6.0 Global Instrumental Earthquake Catalogue^30,31^ and the ANSS Comprehensive Earthquake Catalog^32^. These data sets are used most widely in studies on global seismicity. The magnitude (M) scale in the ISC-GEM ver 6.0 Global Instrumental Earthquake Catalogue is moment magnitude (Mw), whereas the ANSS Comprehensive Earthquake Catalog is a composite catalogue and includes different magnitude scales. We selected events of M 7.0 or larger (Supplementary Data S1 to S4).

We evaluated the statistics corresponding to the period 1904–2015, considering the temporal coverage of the ISC-GEM ver 6.0 Global Instrumental Earthquake Catalogue. We obtained results using (1) global data without any further restriction and (2) global data restricted to subduction zone earthquakes, selecting events within a 200-km-wide band along the trench axes (excluding outer-rise areas) and 100-km deep or shallower (Supplementary Fig. S1). We confirmed that there was no systematic change in the annual number of earthquakes larger than or equal to M 7.0, validating the derivation of statistics for the range of 1904–2015 (Supplementary Fig. S2).

To calculate the probability of successive occurrence of two M8 + earthquakes (${P}_{successive}$), we counted the number of events of an M8 + earthquake followed by another M8 + earthquake that was temporally and spatially proximal (Supplementary Data S5 to S8). We used multiple time frames of 1 day (24 h), 3 days, 1 week, 2 weeks and 3 years. Moreover, an epicentral distance of 500 km was adopted as the threshold. This distance is comparable to the supposed total rupture dimension of the Nankai Trough region. Subsequently, we calculated the probability by dividing the number of successive occurrences by the total number of occurrences of M8 + earthquakes (Supplementary Table S1).

We also calculated the 95% confidence interval and the probability gain for each time frame. The confidence intervals were calculated using the Clopper–Pearson method. The probability gain *G* was obtained by dividing the probability ${P}_{successive}$ by its base rate ${P}_{base}$ for the corresponding duration:

4 $$\begin{array}{c}G= \frac{{P}_{successive}}{{P}_{base}} .\end{array}$$

To determine the ${P}_{base}$, we assumed a Poisson model with an average recurrence interval of 90 years (Supplementary Table S2). The assumed average recurrence interval is consistent with the estimates of the ERC^2^. Note that the probability gain is specific to the Nankai megathrust, and the values for areas with different average recurrence intervals would differ substantially. While the assumption of the Poisson process is common when there is no evidence to the contrary, another applicable model for calculating the base probability is the Brownian Passage Time (BPT) model^44^, which assumes a quasi-periodical earthquake occurrence in the presence of steady stress loading. The BPT model is adopted by the ERC^2^ to calculate the long-term earthquake occurrence probability of major faults in Japan. Adopting the BPT model with the same average recurrence interval of 90 years, fluctuation parameter of 0.24, and the elapsed time since the last earthquake of 75 years leads to a value approximately twice the base rate (half the probability gain). Using a longer average recurrence interval of 120 years, which may be a valid assumption considering the historical records (Section “Probability of successive occurrence from past Nankai megathrust earthquakes”), would lead to a smaller base rate for the BPT model compared with that of the Poisson model. Notably, the estimation of the base rate itself entails significant uncertainty.

To calculate the probability of the successive occurrence of an M7-class earthquake and an M8 + earthquake, we adopted the same procedure with the exception that the threshold for the epicentral distance was set to 160 km (Supplementary Data S9 to S12). This epicentral distance limit was chosen considering the scaling relation between M8 + and M7-class earthquakes. A decrease in the magnitude by one unit corresponds to a decrease in seismic moment by a factor of 10^1.5^, and a decrease in a characteristic length scale by a factor of 10^0.5^, assuming the geometrical similarity between M8 + and M7-class earthquakes. The resulting probabilities obtained by dividing the number of successive occurrences by the total number of M7-class earthquakes are listed in Supplementary Table S3.

The probability gain values calculated using the data in Supplementary Tables S1 to S3 and Eq. (3) are listed in Supplementary Tables S4 and S5.

### Evaluation of past Nankai megathrust earthquakes

Regarding the 1361 Koan earthquake, historical and geological evidence demonstrates the rupture of the Nankai and Tonankai segments. The damage to the Ise Grand Shrine located along the north-eastern coast of the Kii peninsula indicates that the rupture of the Tonankai segment occurred in the same month as that of the Nankai segment^45^, suggesting the possibility of a simultaneous rupture of the two segments or successive rupture of the two segments separated by a time interval of 1 month.

Regarding the 1498 Meio earthquake, the rupture along the Tonankai segment and a part of the Tokai segment is definite, whereas the rupture along the Nankai segment is still under debate^9,14^. Geological evidence is sparse^8^ for the rupture along the Nankai segment; however, evidence of liquefaction attributable to strong earthquake motions was found at archaeological sites on the Shikoku Island^11,34^, along with circumstantial evidence of rupture^35^. The possibility of occurrence of a rupture along the Nankai segment two and a half months before the occurrence of the ruptures along the Tonankai and Tokai segments has been suggested^46^. In summary, in 1498, the Nankai segment probably ruptured simultaneously with the eastern segments, or two and a half months before the rupture of the eastern segments, or did not rupture at all (another strong earthquake could have occurred, causing the liquefaction).

The 1605 rupture is characterized by extensive tsunami damage along the Pacific coast in western Japan and a lack of reports of the damages attributed to the strong ground motions^35,47^. It is inferred from the historical and geological data that the shallow portion of the Nankai and Tonankai segments had ruptured^8,48^, although some researchers question the rupture on the Nankai megathrust and suggest a great earthquake along the Izu-Bonin Trench as the tsunami source^49^.

Numerous studies have been published on the 1707 Hoei, 1854 Ansei Tokai, 1854 Ansei Nankai, 1944 Showa Tonankai, and 1946 Showa Nankai earthquakes, implying greater certainty of the earthquakes occurring after 1707. The 1707 Hoei earthquake was associated with a single rupture along the Nankai and Tonankai segments^6,37^. The 1854 Ansei Tokai earthquake rupturing the Tonankai segment was followed by the Ansei Nankai earthquake rupturing the Nankai segment approximately 30 h later^6,14^. The 1944 Showa Tonankai earthquake was followed by the 1946 Showa Nankai earthquake 2 years and 14 days later, rupturing similar but smaller areas of the Tonankai and Nankai segments, respectively^6,14,17^.

M8 + earthquakes that ruptured only the Tokai or Hyuganada segments are not known, although the Hyuganada segment exhibited M ~ 7.5 earthquakes^14^.

## Supplementary Information

Supplementary Information.

Supplementary Data.

## Data Availability

All data are available in the main text and as Supplementary Information.

## References

[1] Jordan TH (2011). Operational earthquake forecasting: State of knowledge and guidelines for utilization. Ann. Geophys..

[2] Earthquake Research Committee. List of long-term evaluation of earthquake occurrence along major active faults and subduction (in Japanese). Accessed 6 Apr 2022. https://www.jishin.go.jp/evaluation/long_term_evaluation/lte_summary (2022).

[3] Fujiwara H (2006). National seismic hazard maps of Japan. Bull. Earthquake Res. Inst. Univ. Tokyo.

[4] Field EH (2015). Long-term time-dependent probabilities for the third uniform California earthquake rupture forecast (UCERF3). Bull. Seismol. Soc. Am..

[5] Christophersen, A. *et al.* Progress and challenges in operational earthquake forecasting in New Zealand. *2017 New Zealand Society of Earthquake Engineering Conference* (2017).

[6] Ando M (1975). Source mechanisms and tectonic significance of historical earthquakes along the Nankai Trough, Japan. Tectonophysics.

[7] Fujiwara O, Goto K, Ando R, Garrett E (2020). Paleotsunami research along the Nankai Trough and Ryukyu Trench subduction zones—Current achievements and future challenges. Earth-Sci. Rev..

[8] Garrett E (2016). A systematic review of geological evidence for Holocene earthquakes and tsunamis along the Nankai-Suruga Trough, Japan. Earth-Sci. Rev..

[9] Ishibashi K, Satake K (1998). Problems on forecasting great earthquakes in the subduction zones around Japan by means of paleoseismology (in Japanese with English abstract). Zisin.

[10] Kumagai H (1996). Time sequence and the recurrence models for large earthquakes along the Nankai Trough revisited. Geophys. Res. Lett..

[11] Sangawa A (1993). Research on paleoearthquakes using traces discovered at archaeological sites (in Japanese). Quat. Res..

[12] Yokota Y, Ishikawa T, Watanabe S-I, Tashiro T, Asada A (2016). Seafloor geodetic constraints on interplate coupling of the Nankai Trough megathrust zone. Nature.

[13] Hashimoto M (2022). Is the long-term probability of the occurrence of large earthquakes along the Nankai Trough Inflated?—Scientific review. Seismol. Res. Lett..

[14] Earthquake Research Committee. On the Long-Term Evaluation of the Earthquake Activity Along the Nankai Trough (Ver. 2) (in Japanese). Accessed 23 Aug 2022. https://www.jishin.go.jp/main/chousa/13may_nankai/nankai2_shubun.pdf (2013).

[15] Ishibashi K (2004). Status of historical seismology in Japan. Ann. Geophys..

[16] Aida I (1981). Numerical experiments for the tsunamis generated off the coast of the Nankaido district. Bull. Earthq. Res. Inst. Univ. Tokyo.

[17] Sagiya T, Thatcher W (1999). Coseismic slip resolution along a plate boundary megathrust: The Nankai Trough, southwest Japan. J. Geophys. Res..

[18] Ito Y (2013). Episodic slow slip events in the Japan subduction zone before the 2011 Tohoku-Oki earthquake. Tectonophysics.

[19] Kato A (2012). Propagation of slow slip leading up to the 2011 M(w) 9.0 Tohoku-Oki earthquake. Science.

[20] Kato A, Fukuda J, Kumazawa T, Nakagawa S (2016). Accelerated nucleation of the 2014 Iquique, Chile Mw 8.2 Earthquake. Sci. Rep..

[21] Melbourne TI (2002). Precursory transient slip during the 2001 Mw = 8.4 Peru earthquake sequence from continuous GPS. Geophys. Res. Lett..

[22] Ruiz S (2014). Intense foreshocks and a slow slip event preceded the 2014 Iquique Mw 8.1 earthquake. Science.

[23] Socquet A (2017). An 8 month slow slip event triggers progressive nucleation of the 2014 Chile megathrust. Geophys. Res. Lett..

[24] Voss N (2018). Do slow slip events trigger large and great megathrust earthquakes?. Sci. Adv..

[25] Obara K, Kato A (2016). Connecting slow earthquakes to huge earthquakes. Science.

[26] Takagi R, Uchida N, Obara K (2019). Along-strike variation and migration of long-term slow slip events in the western Nankai subduction zone Japan. J. Geophys. Res..

[27] Study Group for the Forecastability of Major Earthquakes along the Nankai Trough. On the forecastability of major earthquakes along the Nankai Trough (in Japanese). Accessed 17 Sept 2022. https://www.bousai.go.jp/jishin/nankai/tyosabukai_wg/pdf/h290825honbun.pdf (2017).

[28] Hashimoto, T. & Yokota, T. Successively occurring large earthquakes in the world - Comparison of real cases with expectations by the space-time ETAS. *Japan Geoscience Union 2019 Meeting* SSS10-P02 (2019).

[29] Cabinet Office of Japan. Guidelines for Formulating Disaster Risk Management Measures based on Various Nankai Trough Earthquake Scenarios (1st edition) (in Japanese). Accessed 3 May 2022. https://www.bousai.go.jp/jishin/nankai/pdf/honbun_guideline2.pdf (2019).

[30] Di Giacomo D, Engdahl ER, Storchak DA (2018). The ISC-GEM earthquake catalogue (1904–2014): Status after the extension project. Earth Syst. Sci. Data.

[31] Storchak DA (2013). Public release of the ISC–GEM global instrumental earthquake catalogue (1900–2009). Seismol. Res. Lett..

[32] U.S. Geological Survey earthquake hazards program. Advanced National Seismic System (ANSS) comprehensive catalog of earthquake events and products. Accessed 18 Apr 2022. https://www.sciencebase.gov/catalog/item/52eab950e4b0444d1ce67917 (2019).

[33] Ishibashi K (1999). Great Tokai and Nankai, Japan, earthquakes as revealed by historical seismology: 1. Review of the events until the mid-14th century (in Japanese with English abstract). J. Geogr. (Chigaku Zasshi).

[34] Sangawa A (2009). A study of paleoearthquakes at archeological sites. Synthesiology.

[35] Usami T, Ishii H, Imamura T, Takemura M, Matsuura R (2013). Materials for Comprehensive List of Destructive Earthquakes in Japan.

[36] Baba T, Tanioka Y, Cummins PR, Uhira K (2002). The slip distribution of the 1946 Nankai earthquake estimated from tsunami inversion using a new plate model. Phys. Earth Planet. Inter..

[37] Furumura T, Imai K, Maeda T (2011). A revised tsunami source model for the 1707 Hoei earthquake and simulation of tsunami inundation of Ryujin Lake, Kyushu, Japan. J. Geophys. Res..

[38] Kanamori H (1972). Tectonic implications of the 1944 Tonankai and the 1946 Nankaido earthquakes. Phys. Earth Planet. Inter..

[39] Kawasumi H (1951). Measure of earthquake danger and expectancy of maximum intensity throughout Japan as inferred from the seismic activity in historical times. Bull. Earthq. Res. Inst..

[40] Tanioka Y, Satake K (2001). Detailed coseismic slip distribution of the 1944 Tonankai Earthquake estimated from tsunami waveforms. Geophys. Res. Lett..

[41] Tanioka Y, Satake K (2001). Coseismic slip distribution of the 1946 Nankai earthquake and aseismic slips caused by the earthquake. Earth Planets Space.

[42] Ogata Y (1992). Detection of precursory relative quiescence before great earthquakes through a statistical model. J. Geophys. Res..

[43] Ogata Y, Zhuang J (2006). Space–time ETAS models and an improved extension. Tectonophysics.

[44] Ellsworth WL (1999). A physically-based earthquake recurrence model for estimation of long-term earthquake probabilities. USGS Open-File Report.

[45] Okuno N, Okuno K (2017). The 1361 Koan earthquake as seen from the damage of Ise Grand Shrine Geku (in Japanese with English abstract). Hist. Earthq. (Rekishi Jishin).

[46] Tsuji, Y. & Ueda, K. Proof of the existence of the 1498 Meio Nankai Earthquake and the date of its occurrence. *Abstracts, Japan Earth and Planetary Science Joint Meeting* 169–169 (1997).

[47] Hatori T (1975). Sources of large tsunamis generated in the Boso, Tokai and Nankai regions in 1498 and 1605. Bull. Earthq. Res. Inst. Univ. Tokyo.

[48] Ando M, Nakamura M (2013). Seismological evidence for a tsunami earthquake recorded four centuries ago on historical documents. Geophys. J. Int..

[49] Ishibashi K. & Harada T. Working hypothesis of the 1605 Great Izu-Bonin Trench Earthquake and the 1614 Nankai Trough earthquake. *Fall Meeting of the Seismological Society of Japan* (2013).

