# Author Correction: High probability of successive occurrence of Nankai megathrust earthquakes

**DOI:** 10.1038/s41598-023-29306-4

**Published:** 2023-02-03

**Authors:** Yo Fukushima, Tomoaki Nishikawa, Yasuyuki Kano

**Affiliations:** 1 International Research Institute of Disaster Science, Tohoku University, Sendai, Japan; 2 Disaster Prevention Research Institute, Kyoto University, Uji, Japan; 3 Earthquake Research Institute, University of Tokyo, Tokyo, Japan

Correction to: *Scientific Reports* https://doi.org/10.1038/s41598-022-26455-w, published online 10 January 2023

The original version of this Article contained errors. In the Abstract,

“Based on global statistics and local earthquake occurrence history, we estimated the probability of a successive occurrence of two M8 or larger earthquakes within 3 years globally and along the Nankai megathrust to be 5.0–18% and 4.3–96%, respectively.”

now reads:

“Based on global statistics and local earthquake occurrence history, we estimated the probability of a successive occurrence of two M8 or larger earthquakes within 3 years globally and along the Nankai megathrust to be 5.3–18% and 4.3–96%, respectively.”

In the Results, under the subheading ‘Probability curve for occurrence of successive Nankai megathrust earthquakes’,

“For example, the probability of an M8 + earthquake occurring within 1 day (24 h) and 1 week after an M8 + earthquake along the Nankai megathrust was 1.4–65% and 2.1–77%, respectively.”

now reads:

“For example, the probability of an M8 + earthquake occurring within 1 day (24 h) and 1 week after an M8 + earthquake along the Nankai megathrust was 1.4–64% and 2.1–77%, respectively.”

In Table 1, in the “Probability (%)” column corresponding to "2 weeks" value,

“2.3–85”

now reads:

“2.3–81”

Additionally, the Acknowledgements section was incomplete.

“This study was funded by the SECOM Science and Technology Foundation. We are grateful to the members of the SECOM Science and Technology Foundation project, especially Motoyuki Kido, Shunichi Koshimura, Ryota Hino, and Yusaku Ohta for fruitful discussion”

now reads:

"This study was funded by the SECOM Science and Technology Foundation. We are grateful to the members of the SECOM Science and Technology Foundation project, especially Motoyuki Kido, Shunichi Koshimura, Ryota Hino, and Yusaku Ohta for fruitful discussion. This study was also funded by the Ministry of Education, Culture, Sports, Science and Technology (MEXT) of Japan, under the Second Earthquake and Volcano Hazards Observation and Research Program.”

The original Article has been corrected.

