# Supplementary material for: High probability of successive occurrence of Nankai megathrust earthquakes
Source: Sci Rep. 2023 Jan 10;13:63. doi: 10.1038/s41598-022-26455-w (PMC9832165; doi:10.1038/s41598-022-26455-w)
Supplement: Supplementary file 1 — Supplementary Information. [file 41598_2022_26455_MOESM1_ESM.pdf]

Supplementary Information for

High probability of successive occurrence of Nankai megathrust earthquakes

Yo Fukushima, Tomoaki Nishikawa, and Yasuyuki Kano

Correspondence to: fukushima@irides.tohoku.ac.jp

This PDF file includes:

Supplementary Figure S1 to S2
Supplementary Tables S1 to S6
Captions for Supplementary Data S1 to S12

Other supplementary materials for this manuscript include the following:

Supplementary_Data.xlsx: Data S1 to S12 in a table format.

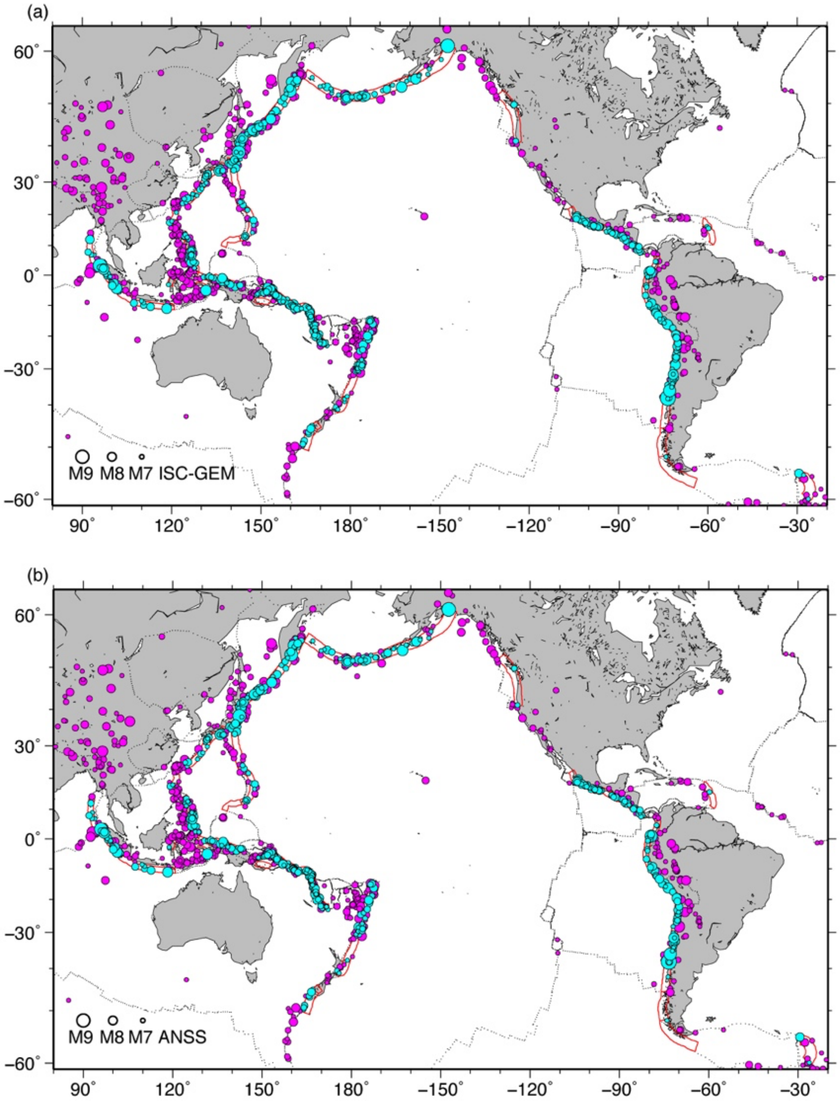

Figure S1. Epicenters of M7 or larger earthquakes used in this study.
(a) ISC-GEM and (b) ANSS catalogs. The cyan circles indicate subduction zone earthquakes within a 200-km-wide band along the trench axes, excluding outer-rise areas and shallower than 100 km. The magenta circles indicate other earthquakes. The red polygons indicate the locations of the subduction zones considered in this study.

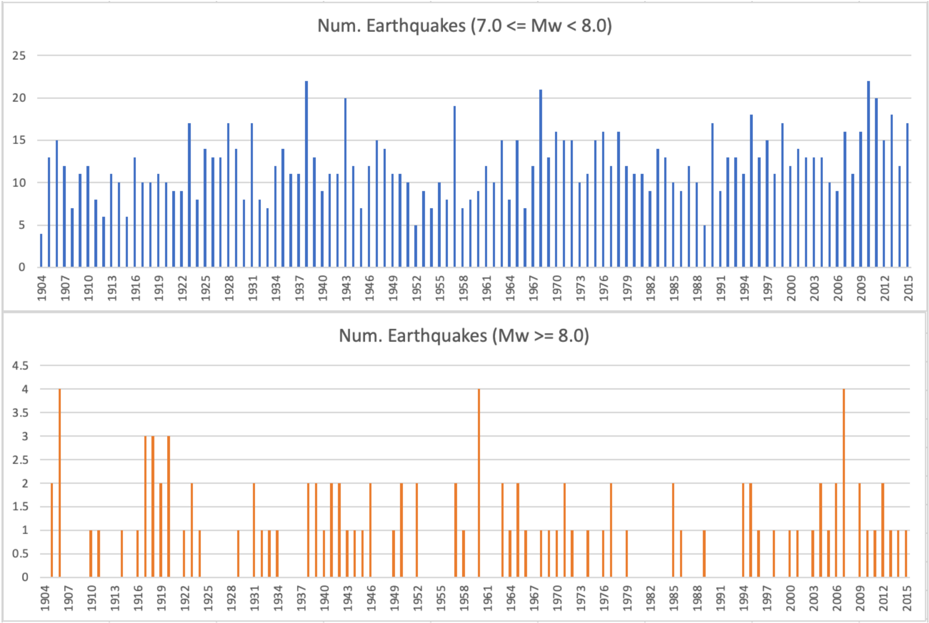

Figure S2. Yearly number of earthquakes in the ISC-GEM catalogue. (Top) Number of earthquakes larger than or equal to Mw 7.0. (Bottom) Number of earthquakes larger than or equal to Mw 8.0.

Table S1. Probability of successive occurrence of M8+ earthquakes

Dataset	Within 24 h	3 days	1 week	2 weeks	3 years
ISC (all)<br>(105 events)	1.9%<br>(2 events)	2.9%<br>(3 events)	2.9%<br>(3 events)	4.8%<br>(5 events)	10%<br>(11 events)
95% CI	0.23%–6.7%	0.59%–8.1%	0.59%–8.1%	1.6%–11%	5.3%–18%
ISC (subduction)<br>(69 events)	1.4%<br>(1 event)	2.9%<br>(2 events)	2.9%<br>(2 events)	4.3%<br>(3 events)	8.7%<br>(6 events)
95% CI	0.037%–7.8%	0.35%–10%	0.35%–10%	0.91%–12%	3.3%–18%
ANSS (all)<br>(92 events)	1.1%<br>(1 event)	2.2%<br>(2 events)	2.2%<br>(2 events)	4.3%<br>(4 events)	9.8%<br>(9 events)
95% CI	0.028%–5.9%	0.26%–7.6%	0.26%–7.6%	1.2%–11%	4.6%–18%
ANSS (subduction)<br>(55 events)	0%<br>(0 event)	1.8%<br>(1 event)	1.8%<br>(1 event)	3.6%<br>(2 events)	9.1%<br>(5 events)
95% CI	0.0%–6.5%	0.046%–9.7%	0.046%–9.7%	0.44%–13%	3.0%–20%

Table S2. Base probability of earthquake occurrence assuming Poisson model with average recurrence interval of 90 years

Time frame	Probability (%)
6 hours	7.6×10^4
12 hours	1.5×10^3
24 hours	3.0×10^3
3 days	9.1×10^3
1 week	2.1×10^2
2 weeks	4.3×10^2
1 month	9.3×10^2
3 years	3.3

Table S3. Probability of successive occurrence of M7-class earthquake and M8+ earthquake

Dataset	Within 24 h	3 days	1 week	2 weeks	3 years
ISC (all) (1,354 events)	0.22% (3 events)	0.37% (5 events)	0.59% (8 events)	0.66% (9 events)	1.7% (23 events)
95% CI	0.046%–0.65%	0.12%–0.86%	0.26%–1.2%	0.30%–1.3%	1.1%–2.5%
ISC (subduction) (611 events)	0.49% (3 events)	0.82% (5 events)	1.3% (8 events)	1.5% (9 events)	3.1% (19 events)
95% CI	0.10%–1.4%	0.27%–1.9%	0.57%–2.6%	0.68%–2.8%	1.9%–4.8%
ANSS (all) (1,252 events)	0.24% (3 events)	0.40% (5 events)	0.40% (5 events)	0.48% (6 events)	1.0% (13 events)
95% CI	0.049%–0.70%	0.13%–0.93%	0.13%–0.93%	0.18%–1.0%	0.55%–1.8%
ANSS (subduction) (545 events)	0.55% (3 events)	0.92% (5 events)	0.92% (5 events)	1.1% (6 events)	1.8% (10 events)
95% CI	0.11%–1.6%	0.30%–2.1%	0.30%–2.1%	0.41%–2.4%	0.88%–3.4%

Table S4. Probability gain calculated for successive occurrence of M8+ earthquakes (Table S1) and base rates (Table S2) for Nankai megathrust

Dataset	Within 24 h	3 days	1 week	2 weeks	3 years
ISC (all)	6.3×10^2	3.1×10^2	1.3×10^2	1.1×10^2	3.2
95% CI	$76–2.2 \times 10^3$	$65–8.9 \times 10^2$	$28–3.8 \times 10^2$	$37–2.5 \times 10^2$	1.6–5.5
ISC (subduction)	4.8×10^2	3.2×10^2	1.4×10^2	1.0×10^2	2.7
95% CI	$12–2.6 \times 10^3$	$39–1.1 \times 10^3$	$17–4.7 \times 10^2$	$21–2.9 \times 10^2$	0.99–5.5
ANSS (all)	3.6×10^2	2.4×10^2	1.0×10^2	1.0×10^2	3.0
95% CI	$9.1–1.9 \times 10^3$	$29–8.4 \times 10^2$	$12–3.6 \times 10^2$	$28–2.5 \times 10^2$	1.4–5.4
ANSS (subduction)	0.0	2.0×10^2	85	85	2.8
95% CI	$0.0–2.1 \times 10^3$	$5.0–1.0 \times 10^3$	$2.2–4.6 \times 10^2$	$10–2.9 \times 10^2$	0.92–6.1
Base rate (%) *	3.0×10^{-3}	9.1×10^{-3}	2.1×10^{-2}	4.3×10^{-2}	3.3

* Values obtained from the ISC-GEM catalog without area restrictions.

Table S5. Probability gain for successive occurrence of M7-class earthquake and M8+ earthquake (Table S3) and the base rates (Table S2) for Nankai megathrust

Dataset	Within 24 h	3 days	1 week	2 weeks	3 years
ISC (all)	73	40	28	16	0.52
95% CI	1.5–2.1 × 10^2	13–94	12–55	7.1–30	0.33–0.77
ISC (subduction)	1.6 × 10^2	90	61	35	0.95
95% CI	33–4.7 × 10^2	29–2.1 × 10^2	27–1.2 × 10^2	16–65	0.57–1.5
ANSS (all)	79	44	19	11	0.32
95% CI	16–2.3 × 10^2	14–1.0 × 10^2	6.1–44	4.1–24	0.17–0.54
ANSS (subduction)	1.8 × 10^2	2.0 × 10^2	85	85	2.8
95% CI	37–5.3 × 10^2	33–2.3 × 10^3	14–1.0 × 10^2	9.5–56	0.27–1.0
Base rate (%) *	3.0 × 10^{-3}	9.1 × 10^{-3}	2.1 × 10^{-2}	4.3 × 10^{-2}	3.3

* Values obtained from the ISC-GEM catalog without area restrictions.

Table S6. Moment magnitude range of the past Nankai megathrust earthquakes estimated from previous studies

Year	Minimum Mw*	Maximum Mw*
1361[A,B]	8.25	8.5
1498 [A,B]	8.2	8.6
1605 [A,B,C]	7.9[#]	8.5
1707 [A,B,D,E,F]	8.4	8.7
1854 (East) [A,B,C,E]	8.4	8.4
1854 (West) [A,B,D,E]	8.4	8.4
1944 [C,E,G,H,I]	8.1	8.2
1946 [D,E,G,H,J,K]	8.1	8.4

A: Kawasumi (1951), B: Usami *et al*. (2013), C: Aida (1981a), D: Aida (1981b), E: Ando (1975), F: Furumura *et al.* (2011), G: Sagiya and Thatcher (1999), H: Kanamori (1972), I: Tanioka and Satake (2001a), J: Tanioka and Satake (2001b), K: Baba *et al*. (2002).

* Rigidity of 50 GPa was assumed in calculating the moment magnitude from the studies that only provided the seismic moment. In the documents A and B, the size of the earthquake is given simply as “magnitude”, which was interpreted in this table as the moment magnitude.

# Kawasumi (1951) estimated the magnitude of the 1605 event to be 7.9. This value, also adopted by Usami *et al*. (2013) in their compilation, was derived from an empirical relationship between the shaking intensity and the magnitude. It has been well known from other studies that the 1605 earthquake was a tsunami earthquake, which suggests that the estimated magnitude 7.9 was a significant underestimate.

Captions for Data S1 to S12

These data in a table format are provided in a single file (Microsoft Excel format) named Supplementary_Data.xlsx.

Data S1. All M 7.0 or larger earthquakes in the ISC-GEM catalog ver 6.0.

Data S2. M 7.0 or larger subduction zone earthquakes in the ISC-GEM catalog ver 6.0, located within a 200-km-wide band along the trench, and 100 km deep or shallower.

Data S3. All M 7.0 or larger earthquakes in the ANSS catalog.

Data S4. M 7.0 or larger subduction zone earthquakes in the ANSS catalog, located within a 200-km-wide band along the trench, and 100 km deep or shallower.

Data S5. Table of M8-M8 successive occurrence in the ISC-GEM catalog.

Data S6. Table of subduction M8-M8 successive occurrence in the ISC-GEM catalog.

Data S7. Table of M8-M8 successive occurrence in the ANSS catalog.

Data S8. Table of subduction M8-M8 successive occurrence in the ANSS catalog.

Data S9. Table of M7-M8 successive occurrence in the ISC-GEM catalog.

Data S10. Table of subduction M7-M8 successive occurrence in the ISC-GEM catalog.

Data S11. Table of M7-M8 successive occurrence in the ANSS catalog.

Data S12. Table of subduction M7-M8 successive occurrence in the ANSS catalog.
